# Two new species of *Nemoura* Latreille and a new combination of *Amphinemura* Ris (Plecoptera, Nemouridae) from the Nanling Mountains Region of China

**DOI:** 10.3897/BDJ.12.e121451

**Published:** 2024-05-23

**Authors:** Ya-Fei Zhu, Abdur Rehman, Yu-Zhou Du

**Affiliations:** 1 College of Plant Protection & Institute of Applied Entomology, Yangzhou University, Yangzhou, 225009, China College of Plant Protection & Institute of Applied Entomology, Yangzhou University Yangzhou, 225009 China; 2 Joint International Research Laboratory of Agriculture and Agri-Product Safety, The Ministry of Education, Yangzhou University, Yangzhou, 225009, China Joint International Research Laboratory of Agriculture and Agri-Product Safety, The Ministry of Education, Yangzhou University Yangzhou, 225009 China

**Keywords:** *Nemoura*, *Amphinemura*, *Indonemoura*, new species, new combination, China

## Abstract

**Background:**

The family Nemouridae, belonging to Plecoptera, comprises 21 genera and over 700 species found globally, with the greatest diversity observed in temperate regions. *Nemoura* Latreille, 1796 and *Amphinemura* Ris, 1902 are two largest genera of the family with the highest diversity in China.

**New information:**

Two new species of *Nemoura* Latreille, 1796 of the family Nemouridae, *Nemouraexterclava* Zhu, Rehman & Du sp. nov. and *Nemouracerciserrata* Zhu, Du & Rehman sp. nov., are described and illustrated from the Nanling Mountains Region in Guangdong Province, southern China. The morphological characteristics of the new species are compared with related taxa. Additionally, the status of *Indonemouravoluta* Li & Yang, 2008, originally from Maoershan National Natural Reserve in Guangxi Zhuang Autonomous Region, was addressed and moved to the genus *Amphinemura* Ris, 1902 on the basis of newly-caught topotypes.

## Introduction

*Nemoura* Latreille, 1796 and *Amphinemura* Ris, 1902 are the two largest genera of Nemouridae in China. Each contains approximately 200 valid species from the Holarctic and Oriental Regions (Baumann 1975, Yang et al. 2015, Yang and Li 2018, DeWalt et al. 2024). Currently, more than 50 species of *Nemoura* and nearly 110 species of *Amphinemura* have been recorded in China (Yang et al. 2015, Li et al. 2016, Chen and Du 2017, Chen and DU 2017, Mo et al. 2017, Li et al. 2018, Qian et al. 2018, Yang and Li 2018, Mo et al. 2019, Chen 2020, Mo et al. 2020c, Mo et al. 2020a, Mo et al. 2020b, Rehman et al. 2021, Teslenko and Palatov 2021, Zhao and Du 2021a, Zhao and Du 2021b, Zhao and Du 2021c, Huo et al. 2022, Mo et al. 2022, Yan et al. 2022, Zhao et al. 2022, Li et al. 2023, Zhao et al. 2023).

The Nanling Mountains Region is located at 24°00′-26°30′N 110°-116°E and serves as the boundary of Guangdong Province, Guangxi Zhuang Autonomous Region, Hunan and Jiangxi Province. It is considered a priority region for biodiversity conservation, housing 19 wildlife natural reserves, such as Maoershan National Natural Reserve in Guangxi Zhuang Autonomous Region, Chebaling National Natural Reserve and Nanling National Forest Park in Guangdong Province. Historically, seven *Nemoura* species have been recorded in the Nanling Mountains Region, including *N.basispina* Li et Yang, 2006, *N.floralis* Li et Yang, 2006, *N.guangdongensis* Li et Yang, 2006, *N.perforata* Li et Yang, 2006 (Yang et al. 2015), *N.cucurbitata* Mo, Wang, Yang & Li, 2020 (Mo et al. 2020a), *N.biplatta* Zhao & Du, 2021 (Zhao and Du 2021a) and *N.alticalcaneum* Mo, Wang & Li, 2022 (Mo et al. 2022).

Herein, two *Nemoura* species from the Nanling Mountains Region in Guangdong Province are described as new to science. Additionally, we provide detailed illustrations of the previously recorded species, Indonemouravoluta Li & Yang, initially placed in *Indonemoura* Baumann, 1975 from Maoershan National Natural Reserve. We propose to transfer this species to *Amphinemura* Ris, 1902 after examining fresh specimens from the type locality. Detailed descriptions of the new species and new images are provided.

## Materials and methods

All examined specimens were collected by hand or net and preserved in 75% ethanol. The terminalia of adults were examined and illustrated using Keyence VHX-5000 system and final images were prepared using Photoshop CS6. All listed specimens are deposited in the Insect Collection of Yangzhou University (ICYZU), Jiangsu Province, China. Morphological terminology of Baumann (1975) and Shimizu (1997) were followed

## Taxon treatments

### Nemoura exterclava

Zhu, Rehman & Du sp. nov.

81B267CE-6B96-5699-BF68-27D5B730DAAC

456508F9-6BE0-4B33-A467-62463C93D191

#### Materials

- **Type status:** Holotype. **Occurrence:** recordedBy: Huo Qing-Bo and Yang Xiao; individualCount: 1; sex: Male; lifeStage: Adult; occurrenceStatus: present; occurrenceID: 1796A96B-A20C-5BCA-B7D2-84076E14E824; **Taxon:** class: Insecta; order: Plecoptera; family: Nemouridae; genus: Nemoura; specificEpithet: *exterclava*; taxonRank: species; scientificNameAuthorship: Zhu, Rehman & Du; **Location:** continent: Asia; country: China; countryCode: CN; stateProvince: Guangdong; locality: Shaoguan City, Nanling National Forest Park, Lion Well,; maximumElevationInMeters: 570 m; decimalLatitude: 24.907204; decimalLongitude: 113.061274; **Identification:** identifiedBy: Zhu Ya-Fei, Abdur Rehman, Du Yu-Zhou; dateIdentified: 10-02-2024; **Event:** year: 2024; month: 1; day: 17; **Record Level:** language: en; institutionCode: ICYZU; basisOfRecord: PreservedSpecimen
- **Type status:** Paratype. **Occurrence:** recordedBy: Wang Xing-Min and Wang Zhi-Jie; individualCount: 1; sex: Male; lifeStage: Adult; occurrenceStatus: present; occurrenceID: 600C7FC4-432E-5CD6-991B-BBA5CD4FC4E7; **Taxon:** class: Insecta; order: Plecoptera; family: Nemouridae; genus: Nemoura; specificEpithet: *exterclava*; taxonRank: species; scientificNameAuthorship: Zhu, Rehman & Du; **Location:** continent: Asia; country: China; countryCode: CN; stateProvince: Guangdong; locality: Nanling, Jiuchongtian; **Identification:** identifiedBy: Zhu Ya-Fei, Abdur Rehman, Du Yu-Zhou; dateIdentified: 10-02-2024; **Event:** year: 2007; month: 1; day: 17; **Record Level:** language: en; institutionCode: ICYZU; basisOfRecord: PreservedSpecimen

#### Description

**Figure 1.**
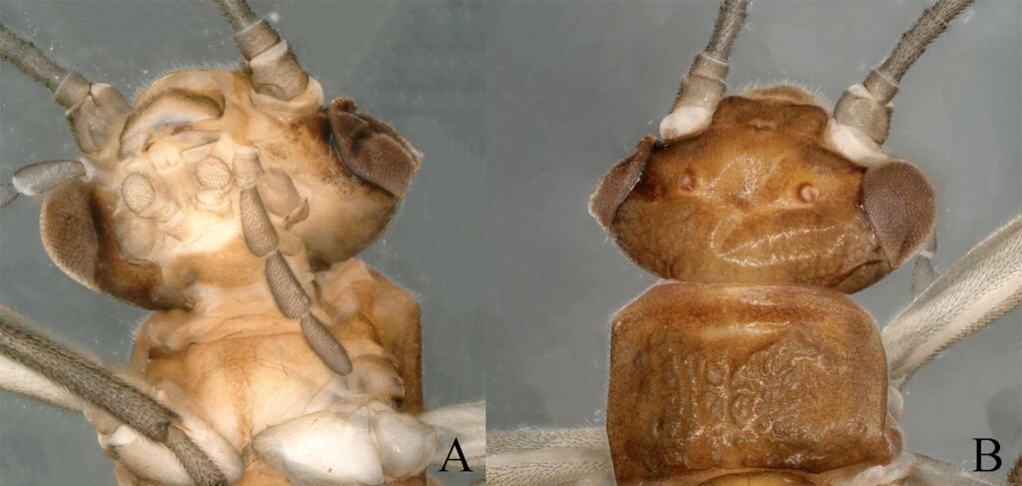
*Nemouraexterclava* Zhu, Rehman & Du sp. nov., male. **A** prothorax, ventral view; **B** head and pronotum, dorsal view.

**Adult habitus**: Head dark brown, antennae brown; head wider than pronotum, compound eyes dark brown, pronotum subquadrate, angles slightly blunt rounded with darker rugosity; wings subhyaline, veins dark brown, legs brown (Fig. 1).

##### Male

**Figure 2.**
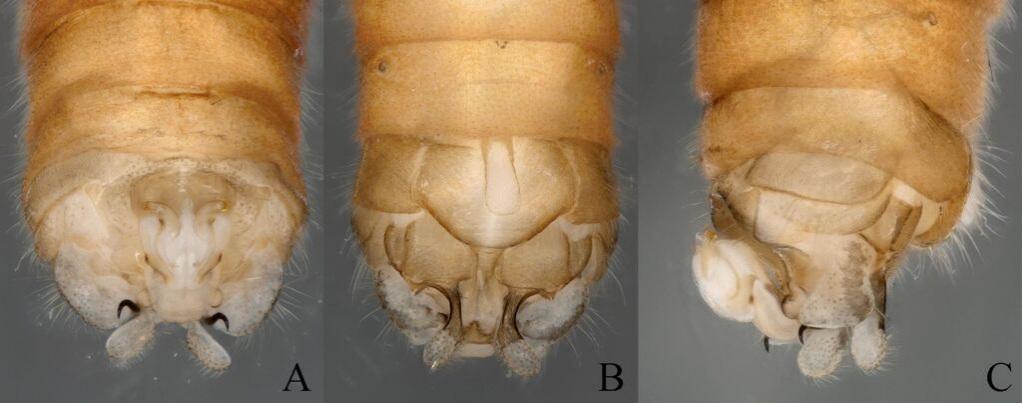
*Nemouraexterclava* Zhu, Rehman & Du sp. nov., male terminalia. **A** dorsal view; **B** ventral view; **C** lateral view.

**Figure 3.**
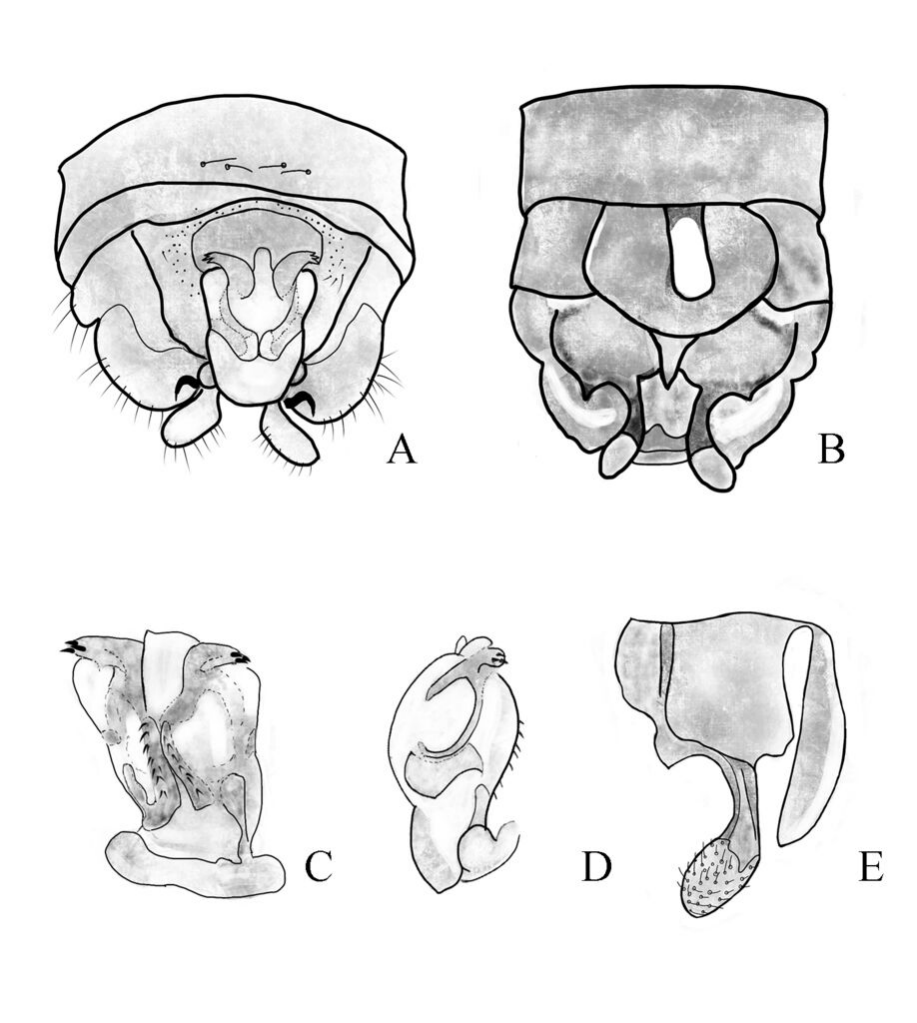
*Nemouraexterclava* Zhu, Rehman & Du sp. nov., male. **A** terminalia, dorsal view; **B** terminalia, ventral view; **C** epiproct, ventral view; **D** epiproct, lateral view; **E** left paraproct, ventral view.

Body length 4.2 mm, fore-wing length 8.2 mm, hind-wing length 7.9 mm. Tergum 9 slightly sclerotised, with concave emargination in the middle and slightly protruding in the middle of posterior margin with several small spines (Fig. 2A and Fig. 3A). Hypoproct broad basally and tapering towards a triangular tip. Vesicle large, length approximately 2× width (Fig. 2B and Fig. 3B). Tergum 10 slightly sclerotised, forming concavity below epiproct, without spines (Fig. 2A and Fig. 3A). Paraproct divided into 2 lobes; Inner lobe slightly sclerotised and narrow anteriorly, spanning approximately 2/3 length of outer lobe. Outer lobe slightly sclerotised and trapeziform at basal part; basal half of outer lobe is broad, sclerotised and becoming slender at 1/2 point; posteromedially becoming narrowed and darker, apex membranous and curving outwards, bearing many hairs, resembling the shape of golf club head (Fig. 3B and Fig. 3E). Epiproct nearly rectangular, trifurcate structure and 2× longer than width. Dorsal sclerite broad basally, extending dorsolaterally, completely covering lateral aspects of epiproct. Epiproct apex pointed dorsally, slightly swollen and has a cone-shaped projection; medially two prongs arise on lateral sides from middle of epiproct and curved outwards, bearing 3 teeth at apex. Lateral arms distinctly sclerotised and run along lateral sides of epiproct (Fig. 3A-C and Fig. 3D). Ventral sclerite darkly sclerotised, broad basally, with blunt rounded tip medially and pair of parallel ridges, armed with two rows of seven spines ventrally. The ridges extend inwards and upwards to dorsal surface and longer than median part (Fig. 3 B). Cerci slightly sclerotised, curved in middle and perpendicular to the body, with scimitar-like black spine at end, which is more distinct in dorsal view (Figs 2, 3).

##### Female

Unknown

#### Etymology

The name refers to the club-shaped process at the apical half of the outer paraproct lobe. *Exter* means outer, while *clava* means club.

#### Distribution

China (Guangdong Province).

#### Taxon discussion

**Figure 4.**
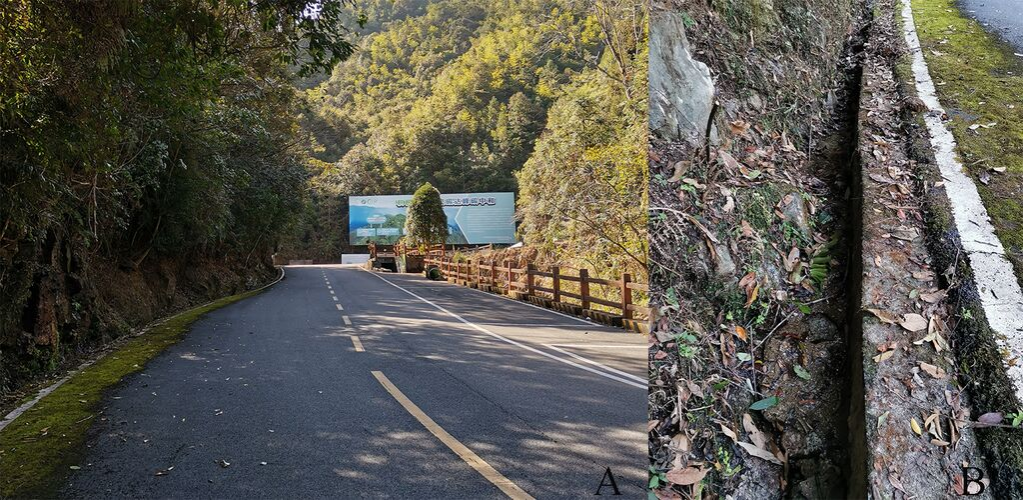
Habitat of *Nemouraexterclava* Zhu, Rehman & Du sp. nov. in Nanling National Forest Park, Lion Well, Guangdong Province, China (Photograph by Huo Qing-Bo).

The new species belongs to the *ovocercia* group and it is most similar to the Nanling Mountains species *Nemouraalticalcaneum* Mo, Wang & Li, 2020 in the epiproct. It can be distinguished from *N.alticalcaneum*, based on the following characteristics: the outer lobe, resembling the shape of a golf club head (Fig. 3E). Epiproct ventral sclerite extends to the dorsal surface, forming two prong-like protrusions longer than median part, each bearing three thick spines apically, in dorsal view (Fig. 3A). Cerci are folded in the middle, bearing long sclerotised prong at the apex (Fig. 3E). In *N.alticalcaneum*, the paraproct has a complex outer lobe, apically with a high-heeled process. The ventral sclerite also extends to the dorsal surface, forming two horn-like protrusions, albeit shorter than the median part, with the apex featuring two blunt teeth, as seen in dorsal view (Mo et al. 2022). Whereas in this new species, the epiproct apex lacks strong serrations on the dorsal margin and the cerci are complex, with a scimitar-like black spine at the end, which is significantly different from recorded speceies. The new species was collected from the road side in Nanling National Forest Park (Fig. 4).

### Nemoura cerciserrata

Zhu, Du & Rehman sp. nov.

E548F8FB-93F4-5926-B813-558D476F87B5

9772E2EB-8E8D-4C80-83D3-304D0CEBB03B

#### Materials

- **Type status:** Holotype. **Occurrence:** individualCount: 1; sex: Male; lifeStage: Adult; occurrenceStatus: present; occurrenceID: 6BB7C443-9554-526F-A0E8-381294D03443; **Taxon:** class: Insecta; order: Plecoptera; family: Nemouridae; genus: Nemoura; specificEpithet: *cerciserrata*; taxonRank: species; **Location:** continent: Asia; country: China; countryCode: CN; stateProvince: Guangdong; locality: Shaoguan City, Shixing county, Chebaling Nature Reserve; decimalLatitude: 24.711731; decimalLongitude: 114.236787; **Identification:** identifiedBy: Zhu Ya-Fei, Abdur Rehman, Du Yu-Zhou; **Event:** year: 2024; month: 1; day: 15,; **Record Level:** institutionCode: ICYZU; basisOfRecord: PreservedSpecimen
- **Type status:** Paratype. **Occurrence:** individualCount: 1; sex: Male; lifeStage: Adult; occurrenceStatus: present; occurrenceID: 29C5728A-30CD-5A41-9352-E82704EF2766; **Taxon:** class: Insecta; order: Plecoptera; family: Nemouridae; genus: Nemoura; specificEpithet: *cerciserrata*; taxonRank: species; **Location:** continent: Asia; country: China; countryCode: CN; stateProvince: Guangdong; locality: Shaoguan City, Shixing county, Chebaling Nature Reserve; decimalLatitude: 24.711731; decimalLongitude: 114.236787; **Identification:** identifiedBy: Zhu Ya-Fei, Abdur Rehman, Du Yu-Zhou; **Event:** year: 2022; month: 1; day: 31; **Record Level:** institutionCode: ICYZU; basisOfRecord: PreservedSpecimen

#### Description

**Figure 5.**
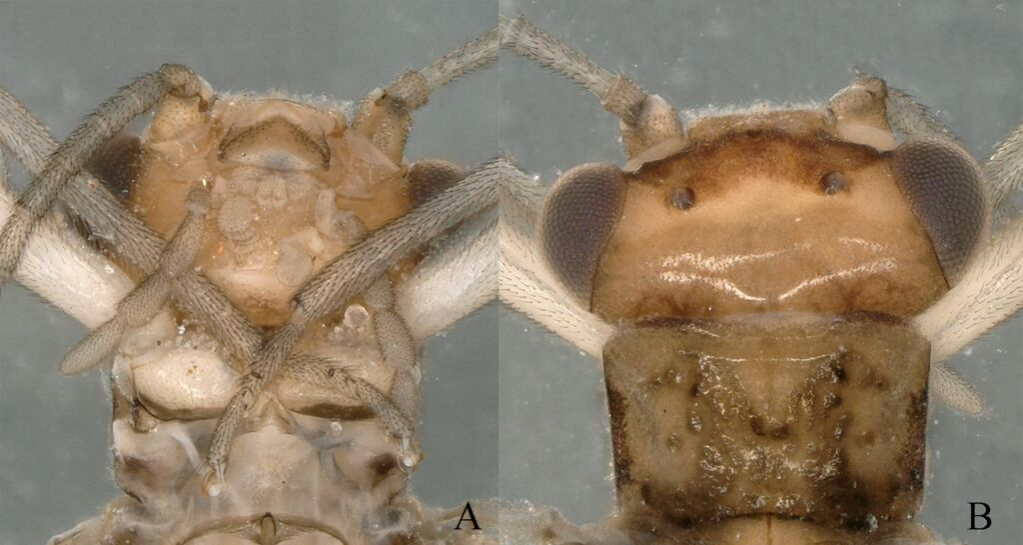
*Nemouracerciserrata* Zhu, Du & Rehman sp. nov., male. **A** prothorax, ventral view; **B** head and pronotum, dorsal view.

**Adult habitus**: Head dark brown, wider than pronotum; antennae pale brown; compound eyes dark brown. Pronotum subquadrate, angles slightly blunt rounded with darker rugosity. Wings subhyaline, veins dark brown. Legs pale brown (Fig. 5).

##### Male

**Figure 6.**
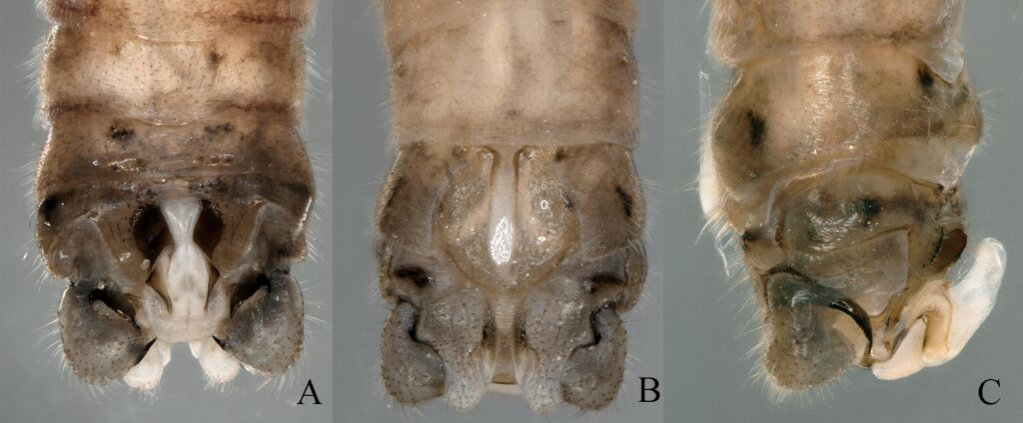
*Nemouracerciserrata* Zhu, Du & Rehman sp. nov., male terminalia. **A** dorsal view; **B** ventral view; **C** lateral view.

**Figure 7.**
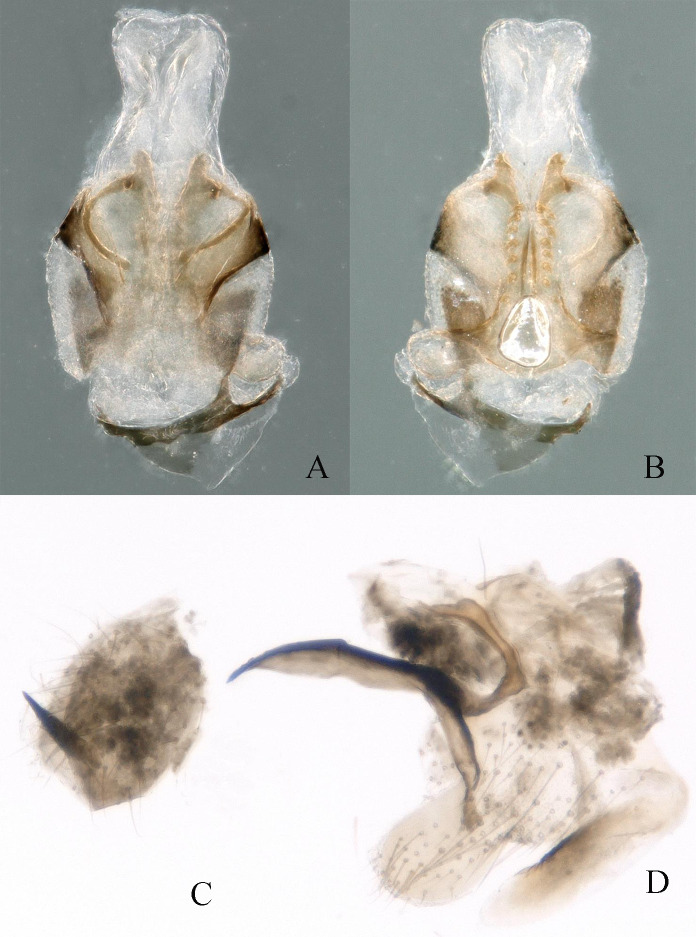
*Nemouracerciserrata* Zhu, Du & Rehman sp. nov. Male, **A** epiproct, dorsal view. **B** epiproct, ventral view; **C** apical part of the cercus with its terminal spine; **D** basal part of the cercus with its strong spur and paraproct.

**Figure 8.**
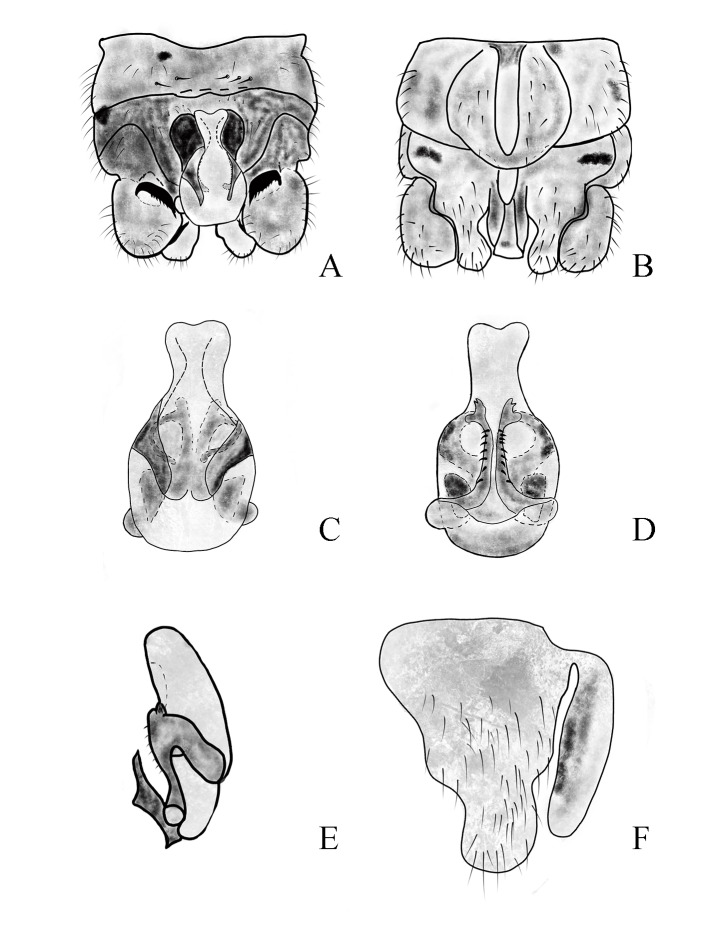
*Nemouracerciserrata* Zhu, Du & Rehman sp. nov. male. **A** Male terminalia dorsal view; **B** Male terminalia ventral view; **C** epiproct, dorsal view; **D** epiproct, ventral view; **E** epiproct, lateral view; **F** left paraproct, ventral view.

**Figure 9.**
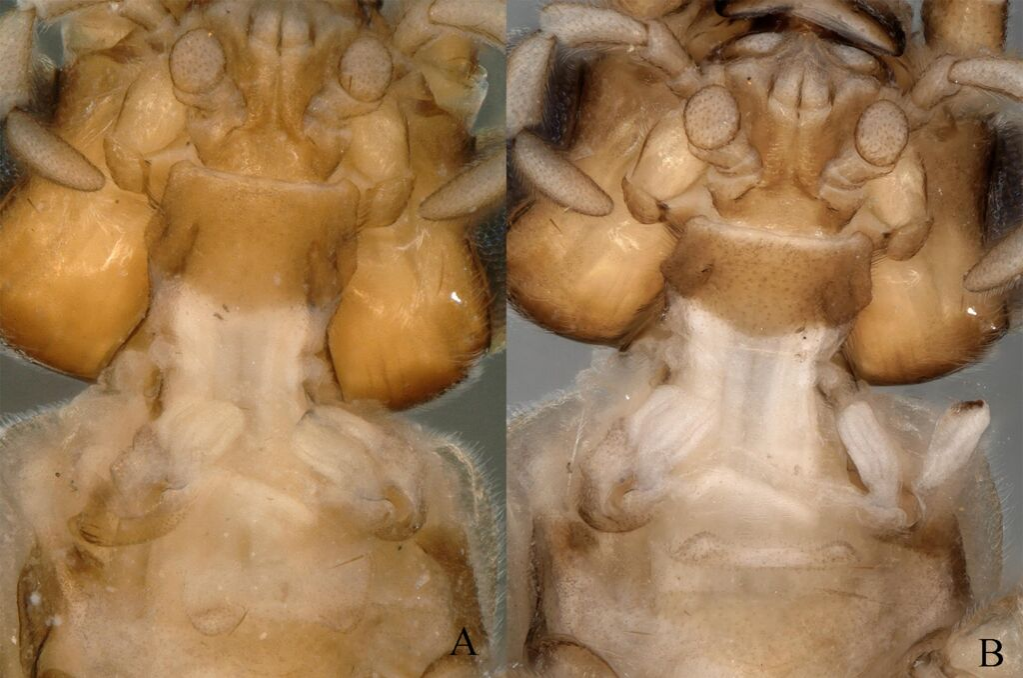
*Amphinemuravoluta* (Li & Yang, 2008), comb. nov. male. **A** prothorax, ventral view; **B** cervical gills, ventral view.

Body length 4.5 mm, fore-wing length 8.5 mm, hind-wing length 8.2 mm. Tergum 9 slightly sclerotised, with concave emargination in the centre and with several small hairs along posterior margin (Fig. 6A and Fig. 8A). Hypoproct broad basally and tapered towards blunt rounded tip. Vesicle rod-like, length approximately 3× width (Figs. 7B and 9B). Tergum 10 strongly sclerotised, forming concavity below epiproct (Fig. 6A and Fig. 8A). Paraproct divided into two lobes: inner lobe sclerotised, shallow arcuate shape and approximately 3/4 length of outer lobe. Outer lobe slightly sclerotised presenting triangular resemblance at base. The outer lobe narrows at halfway point and forms vesicle-like structure at tip (Fig. 6B, Fig. 7C and Fig. 8F). Epiproct has membranous heart-shaped process at the top, 2× longer than width. Dorsal sclerite broad basally, extending dorsolaterally, completely covering lateral aspects of epiproct and part of ventral aspect (Fig. 6A and Fig. 7A). Lateral arms distinctly sclerotised and run along lateral sides of epiproct. Ventral sclerite of epiproct darkly sclerotised, broad basally, forming pair of parallel ridges with 7-9 spines ventrally; these ventral ridges extend upwards to form an antler-like protrusion with two small blunt spines on tip (Fig. 7B and Fig. 8D). Cerci slightly sclerotised with two spines, with strong, spur in the middle and bears sclerotised black pointed spur at the end (Figs 6, 7).

#### Diagnosis

##### Female

Unknown

#### Etymology

The latin *cerciserrata* refers to a strong, curving spur in the middle of the cerci. *Serrata* means toothed, like a saw.

#### Distribution

China (Guangdong Province).

#### Taxon discussion

The new species belongs to the *ovocercia* group and it is most similar to the *Nemouralongistyla* Zhao, Rehman & Du, 2023, as it possesses a comparable epiproct with elongated protrusions at the apex (*Zhao et al. 2023*). It can be distinguished from the latter by the following characteristics: in *Nemoura* sp. nov., the ventral sclerite of epiproct extends upwards to form an antler-like protrusion, with two small blunt spines on the tip. The features are not distinctly visible in the dorsal view (Figs 7, 8), while the ventral sclerite extends upwards to form a horn-like protrusion and the apex has three small teeth in *N.longistyla*, which are more distinct in the lateral view. In addition, there is a strongly sclerotised sawing spine in the middle of the cerci, which is significantly different from *N.longistyla* (Figs 6, 7, 8).

### Amphinemura voluta

(Li & Yang, 2008), comb. nov

38A7A4B0-AC8F-5696-B02E-4E6CE364A971

https://plecoptera.speciesfile.org/otus/892272/overview

*Indonemouravoluta* Li & Yang, 2008 - Li and Yang 2008: 100.

#### Materials

- **Type status:** Other material. **Occurrence:** recordedBy: Unknown; sex: 1; lifeStage: Adult; occurrenceStatus: present; occurrenceID: 80B19B96-02DC-574B-AE80-A79A0B636131; **Taxon:** phylum: Arthropoda;; class: Insecta; order: Plecoptera; family: Nemouridae; genus: Amphinemur; specificEpithet: voluta; scientificNameAuthorship: Li & Yang (2008); **Location:** continent: Asia; country: China; countryCode: CN; locality: Guangxi Zhuang Autonomous Region. Maoershan National Natural Reserve; **Identification:** identifiedBy: Abdur Rehman, Zhu Ya-Fei, Du Yu-Zhou; dateIdentified: 10-02-2024; **Event:** year: 2020; month: 8; day: 29; **Record Level:** language: en; institutionCode: ICYZU; basisOfRecord: PreservedSpecimen

#### Description

**Figure 10.**
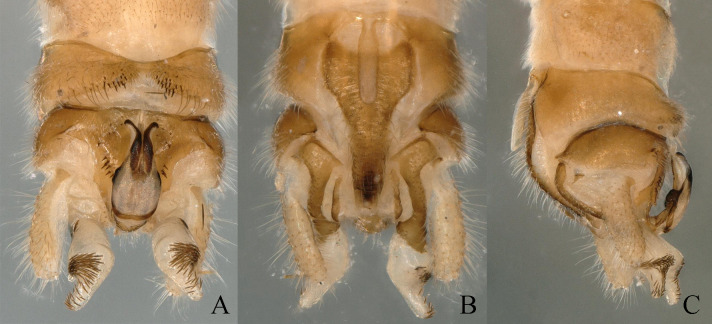
*Amphinemuravoluta* (Li & Yang, 2008), comb. nov. Male terminalia, **A** dorsal view; **B** ventral view; **C** lateral view.

**Male**: Cervical gills shrunk into ball (Fig. 9). Hypoproct broad basally and tapered towards tip. Vesicle slender, length approximately 3× width (Fig. 10B). Tergum 10 weakly sclerotised, with rather large median concavity bearing two groups of tiny black spines along lateral margin. Epiproct trifurcate distally; dorsal sclerite divided into pair of heavily sclerotised, spine-like lateral processes with rounded ventral projection medially (Fig. 11C), distinctly curved, outwards subapically, bearing many spines apically (Fig. 10). Paraproct divided into three lobes: outer lobe reduced and knob-like; median lobe well developed, mostly sclerotised, strongly curved upwards, with bundle of spirally-arranged spines at membranous tip (Fig. 10); inner lobe weakly sclerotised, about 1/3 length of the median lobe, with triangular tip (Li and Yang 2008).

#### Distribution

China (Guangxi Zhuang Autonomous Region).

#### Taxon discussion

This species was collected from Maoershan National Natural Reserve, Guangxi Zhuang Autonomous Region; the same collection sites were recorded in Li and Yang (2008). The cervical gills are shrunk into a ball, which is difficult to distinguish. After slight pressing, it can be seen that there is a cervical gill on each side of the mid-line, one inside and one outside of lateral cervical sclerites and each cervical gill has five branches (Fig. 10B). In addition, the structures of tergum 9, tergum 10, hypoproct, epiproct and paraproct in this specimen are consistent with those described by Li and Yang (2008). Examining fresh specimens from the type locality, we propose to transfer this species to *Amphinemura* Ris, 1902. This species has rather exceptional characteristics; very elongated sausage-shaped cerci while most species of the genus *Amphinemura* have rather short cerci, except in several Asian *Amphinemura* (e.g. *A.mamillata*, *A.nanlingensis* and *A.chui*). Additionally, we consider this species as belonging the *A.sinensis* species group.

## Discussion

The Nanling Mountains Region is a priority area for biodiversity conservation, where the two new species have been discovered: *Nemouraexterclava* Zhu, Du & Rehman sp. nov. from Nanling National Forest Park in Guangdong Province and *Nemouracerciserrata* Zhu, Du & Rehman sp. nov. from Chebaling Nature Reserve in Guangdong Province. While some species bear similarities, these two species are considered new to science due to their distinct morphological differences or characteristics. Given the close distribution of certain similar species, molecular methods could be considered to confirm the validity of new taxa in the future. Furthermore, we propose transferring *Indonemouravoluta* Li & Yang to *Amphinemura* Ris, 1902 after examining fresh specimens from the type locality.

## Supplementary Material

XML Treatment for Nemoura exterclava

XML Treatment for Nemoura cerciserrata

XML Treatment for Amphinemura voluta
